# Beyond the Waitlist: Addressing Structural Racism and Age-Specific Barriers to Equity in Kidney Transplantation

**DOI:** 10.1016/j.ekir.2025.05.043

**Published:** 2025-06-03

**Authors:** Abd A. Qannus, Bekir Tanriover

**Affiliations:** 1 Division of Nephrology, Department of Internal Medicine, The University of Arizona, COM-Tucson, Arizona, USA

See Clinical Research on Page 2766

Kidney transplantation is the preferred treatment for end-stage kidney disease because of its survival and quality-of-life benefits. Despite considerable efforts, including the introduction of the kidney allocation system^1^ in 2014 aimed at reducing disparities in deceased donor kidney transplantation (DDKT), significant racial inequities persist. Black patients had a substantially lower likelihood of being listed for transplantation (13% lower) and faced even greater disparities after being listed, with a 39% lower likelihood of receiving a transplant compared to their White counterparts.^2^ These disparities were particularly severe for living donor kidney transplantation (LDKT) and preemptive transplantation; and whereas kidney allocation system reforms slightly improved equity in DDKT for Black patients, significant disparities remained for LDKT and preemptive transplantation. Contributing factors identified include systemic issues such as reduced health care access,^3^ knowledge gaps,^4^ provider biases,^5^ socioeconomic disadvantages,^6^ and neighborhood conditions.^7^ Moreover, the COVID-19 pandemic further intensified these inequities by disrupting health care practices, especially in LDKT.

In the study, “Divergent Trends by Patient Age in Racial Disparities in Kidney Transplant Access,” published in Kidney International Reports, Buford *et al.*^8^ provide important insights into these dynamics. Using comprehensive data from the United States Renal Data System, the authors examine temporal trends in racial disparities from 2005 to 2019 across 3 key outcomes: waitlisting, LDKT, and DDKT, uniquely stratifying their analysis by age groups. The study uncovers a nuanced landscape of progress and persistent challenges. Encouragingly, the authors report significant improvements in access to DDKT among Black adults who were already waitlisted. Initially, Black adults were 8% less likely to receive a DDKT than White adults during 2005 to 2009; by 2015 to 2019, however, this trend reversed significantly, with Black adults becoming 28% more likely to receive a DDKT. This positive shift suggests that recent policy changes, notably the kidney allocation system reforms, have contributed meaningfully to improving equity among those already on the waitlist. Nevertheless, the study also highlights persistent and worsening disparities, particularly at earlier transplantation stages. Most concerning is the lack of improvement in waitlisting disparities. Among young adults aged 18 to 29 years, the disparity increased notably from a 26% lower likelihood for Black adults in 2005 to 2009 to a 32% lower likelihood by 2015 to 2019. This growing disparity is especially troubling given the profound long-term benefits that younger patients derive from early transplantation. Similarly, the disparities in access to LDKT remained markedly unchanged and persistently high throughout the study period. Black adults consistently remained 63% to 66% less likely than their White counterparts to receive a living donor transplant. Notably, the disparity widened significantly among older adults aged 65 to 80 years, from 51% less likely in 2005 to 2009 to 62% less likely in 2015 to 2019. These findings underscore the need for targeted interventions addressing early-stage barriers and improving living donor transplantation access specifically for Black patients.

The methodological and substantive contributions of this study are significant. Stratifying results by age highlights disparities that aggregated data typically obscure, emphasizing the need for tailored, demographic-specific interventions. In addition, the use of a robust national dataset enhances the generalizability and statistical power of the findings. The longitudinal approach of this research provides valuable insights into the evolving effects of significant policy reforms, offering a comprehensive evaluation of how equity in kidney transplantation has progressed over time.

Nevertheless, as with any large data research, the study has limitations, some acknowledged by the authors. Potential residual confounding exists due to unmeasured variables, including the quality of transplant education, specific evaluation practices at transplant centers, and detailed individual-level socioeconomic factors such as income or educational attainment. Furthermore, the study exclusively examines non-Hispanic Black and non-Hispanic White populations, limiting generalizability to other racial or ethnic groups such as Hispanic, Asian, or Indigenous populations. The exclusion of patients receiving preemptive and repeat transplants likely leads to an underestimation of actual disparities, given the higher rates of preemptive transplantation among White patients. The reliance on CMS-2728 data introduces potential measurement error, particularly if patient characteristics change between the initiation of kidney replacement therapy and eventual waitlisting or transplantation. In addition, using staff-assigned race, although reflective of real-world perceptions and biases, introduces further methodological complexities.

A broader, critical examination of how race is conceptualized in transplantation disparities research is necessary. While appropriately describing race as a social construct indicative of structural inequities rather than biological differences, continued reliance on race as a categorical analytical variable requires careful contextualization. Observed disparities in transplantation outcomes such as lower waitlisting rates and reduced access to LDKT among Black patients are rooted in structural racism and cumulative disadvantage, rather than inherent racial characteristics or individual-level behaviors. Future research would benefit significantly from integrating detailed social determinants of health data, leveraging resources such as the Agency for Healthcare Research and Quality^9^ social determinants of health database (including 5 key social determinants of health domains, namely social, economic, education, physical infrastructure, and health care context). Such comprehensive, neighborhood-level analyses would enable a deeper understanding of the structural mechanisms driving these disparities. Emphasizing upstream socioeconomic determinants and systemic inequities, rather than narrowly focusing on race as an isolated explanatory factor, is crucial to directing interventions effectively and avoiding misinterpretation of race as a biological determinant.

In addition, interventions to address LDKT disparities must transcend educational outreach alone. Comprehensive programs should directly address financial barriers for potential donors, expand paired donation and donor chain programs, offer culturally responsive counseling, and enhance the broader support systems necessary for successful living donation. For younger Black adults, targeted strategies should focus explicitly on the unique challenges encountered during the critical transition from pediatric to adult nephrology care, including insurance coverage gaps, cognitive and developmental barriers, and historically rooted medical mistrust.

Buford *et al.* convey a vital message: achieving equity in kidney transplantation requires interventions earlier in the patient pathway, beyond the current emphasis on improving DDKT access for those already waitlisted. The greatest challenges lie in systematically addressing barriers at the referral and evaluation stages to ensure equitable early access, particularly for Black patients at both ends of the age spectrum.

Ultimately, this study highlights the imperative for intersectional, targeted policy reform, and systemic investments informed by structural understandings of inequity. Addressing root causes rather than merely treating symptoms of inequity is essential for achieving lasting, meaningful progress toward equity in kidney transplantation.

## Disclosure

All the authors declared no competing interests.
